# Delineating colorectal cancer distribution, interaction, and risk prediction by environmental risk factors and serum trace elements

**DOI:** 10.1038/s41598-020-75760-9

**Published:** 2020-10-29

**Authors:** Azmawati Mohammed Nawi, Siok Fong Chin, Luqman Mazlan, Rahman Jamal

**Affiliations:** 1 Department of Community Health, Faculty of Medicine, UKM Medical Center, Universiti Kebangsaan Malaysia, Jalan Yaacob Latif, Bandar Tun Razak, 56000 Cheras, W. Persekutuan Malaysia; 2 Medical Molecular Biology Institute (UMBI), Universiti Kebangsaan Malaysia (UKM), Jalan Yaacob Latif, Bandar Tun Razak, 56000 Cheras, W. Persekutuan Malaysia; 3 Department of Surgery, UKM Medical Center, UKM, Cheras, Malaysia

**Keywords:** Cancer, Biomarkers, Risk factors

## Abstract

The burden of colorectal cancer (CRC) is increasing worldwide especially in developing countries. This phenomenon may be attributable to lifestyle, dietary and environmental risk factors. We aimed to determine the level of 25 trace elements, their interaction with environmental risk factors, and subsequently develop a risk prediction model for CRC (RPM CRC). For the discovery phase, we used a hospital-based case–control study (CRC and non-CRC patients) and in the validation phase we analysed pre-symptomatic samples of CRC patients from The Malaysian Cohort Biobank. Information on the environmental risk factors were obtained and level of 25 trace elements measured using the ICP-MS method. CRC patients had lower Zn and Se levels but higher Li, Be, Al, Co, Cu, As, Cd, Rb, Ba, Hg, Tl, and Pb levels compared to non-CRC patients. The positive interaction between red meat intake ≥ 50 g/day and Co ≥ 4.77 µg/L (AP 0.97; 95% CI 0.91, 1.03) doubled the risk of CRC. A panel of 24 trace elements can predict simultaneously and accurate of high, moderate, and low risk of CRC (accuracy 100%, AUC 1.00). This study provides a new input on possible roles for various trace elements in CRC as well as using a panel of trace elements as a screening approach to CRC.

## Introduction

The incidence and mortality of colorectal cancer (CRC) vary widely from country to country^1^. The incidence and mortality rates are still increasing in low- and middle-income countries^2^, while developed countries such as the United States show a decreasing trend^3^. In Asia, environmental factors and the low public awareness of CRC screening within the different ethnicities and cultures may contribute to the increasing trend in incidence^4^. Among the screening tests for detecting CRC, colonoscopy has a sensitivity of more than 90%, with an estimated 59% reduction in risk of death from CRC^5^. However, it is an invasive procedure and costly, especially in countries that do not provide free population screening. A blood-based marker, which would be relatively inexpensive to measure, could increase screening compliance and be cost-effective^6^.

In recent years, there has been great interest in the analysis of trace elements (TEs) for use as biomarkers due to the diverse roles that TEs play in the various biochemical and physiological processes. The levels of arsenic (As), copper (Cu), cobalt (Co), nickel (Ni), Magnesium (Mg) and lead (Pb) have been reported to be high in several cancers and are believed to contribute to cancer development^7–11^. There are few studies looking at the multi-element approach and most of them have used the atomic absorption spectroscopy approach and not the ICPMS method. There is a need for a multi-element approach to analyse the various TEs in blood samples The levels of TEs in the human body vary according to different environmental exposures and are influenced by diet intake^12,13^, smoking status^14^, and obesity^15^.

Most risk prediction models (RPMs) for CRC use information on non-modifiable (i.e., age, sex) and modifiable environmental factors (i.e., lifestyle, clinical data)^16^. RPMs that use information on environmental factors with the inclusion of TE levels are lacking. An RPM that can identify high-risk groups would be helpful and cost-beneficial and enable the development of focused preventive strategies.

The objectives of this study were to: (i) determine, compare and classify TE levels among CRC cases and controls, (ii) determine the interaction between serum TEs and environmental factors, and (iii) develop and validate a CRC RPM using TE levels and environmental factors.

## Results

### Comparative analysis of environmental risk factors and serum TE levels between CRC and non-CRC patients

**Figure 1:**
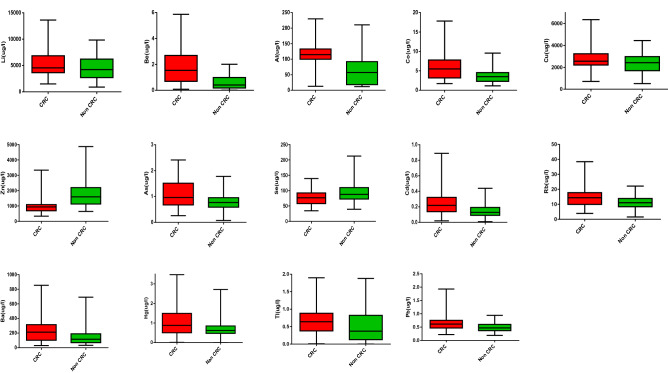
Selected 14 trace elements with significant difference concentration among CRC and non-CRC patients.

The environmental risk factors between the CRC and non-CRC samples were significantly different for sex, smoking status, physical activity, obesity, and between red meat and white meat intake. Most patients with CRC were men (61.8%) while women outnumbered the men in those without CRC (52.9%). Among the CRC patients, 48% were smokers, 66% were overweight and obese, 59.8% were physically inactive, 56.9% had higher red meat intake and 53.9% had lower white meat intake. Fourteen TEs (Li, Be, Al, Co, Cu, Zn, As, Se, Cd, Rb, Ba, Hg, Tl, Pb) showed significant mean/median differences between the patients with and without CRC. Patients with CRC had lower Zn and Se levels but higher Li, Be, Al, Co, Cu, As, Cd, Rb, Ba, Hg, Tl, and Pb levels compared to patients without CRC (Fig. 1).

### Linkage analysis of trace elements identifies disturbed clustering in colorectal cancer

**Figure 2:**
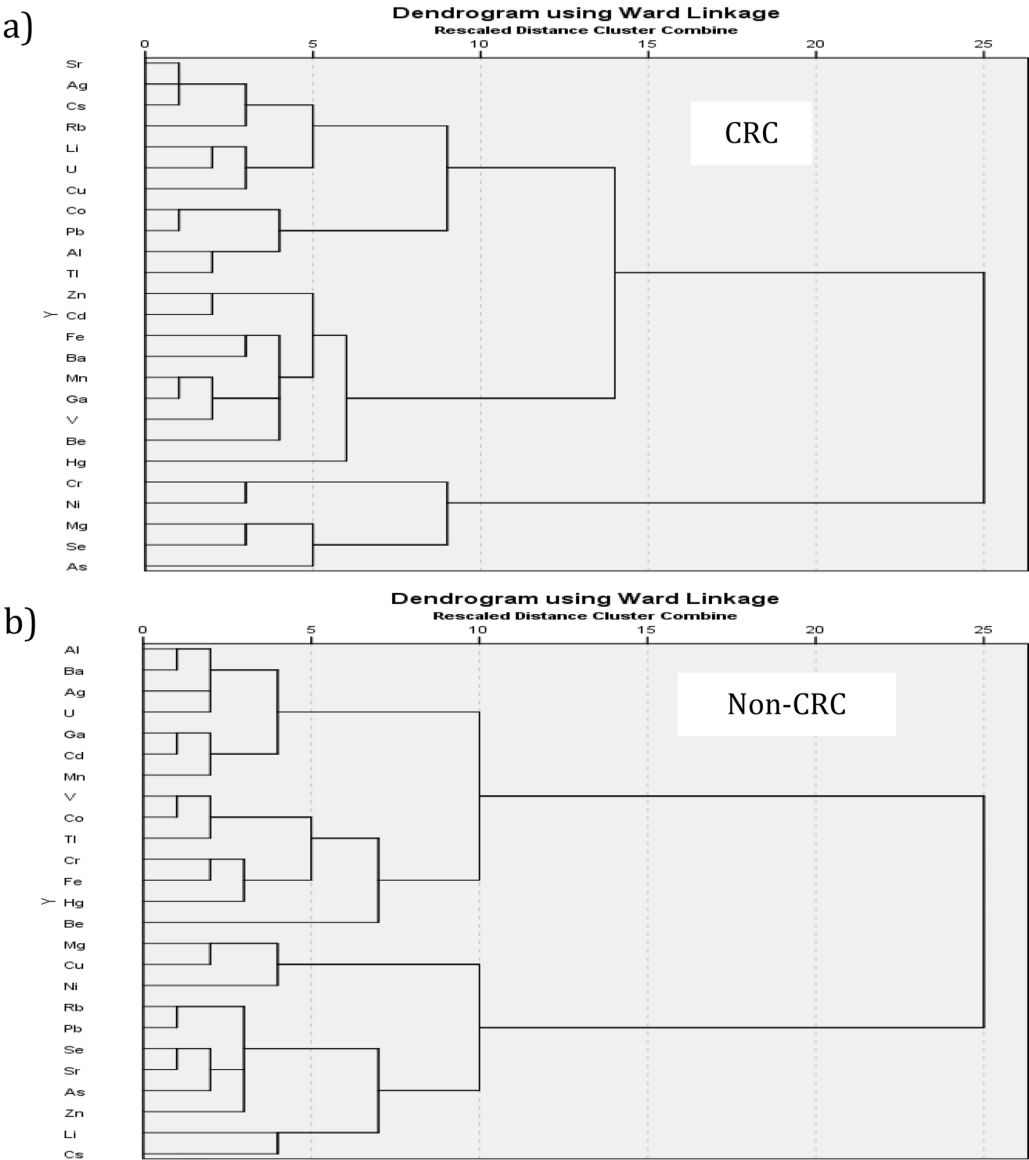
Dendogram using Ward Linkage for 25 trace elements. Different cluster patterns were observed among (**a**) CRC patients, (**b**) non-CRC patients.

**Figure 3:**
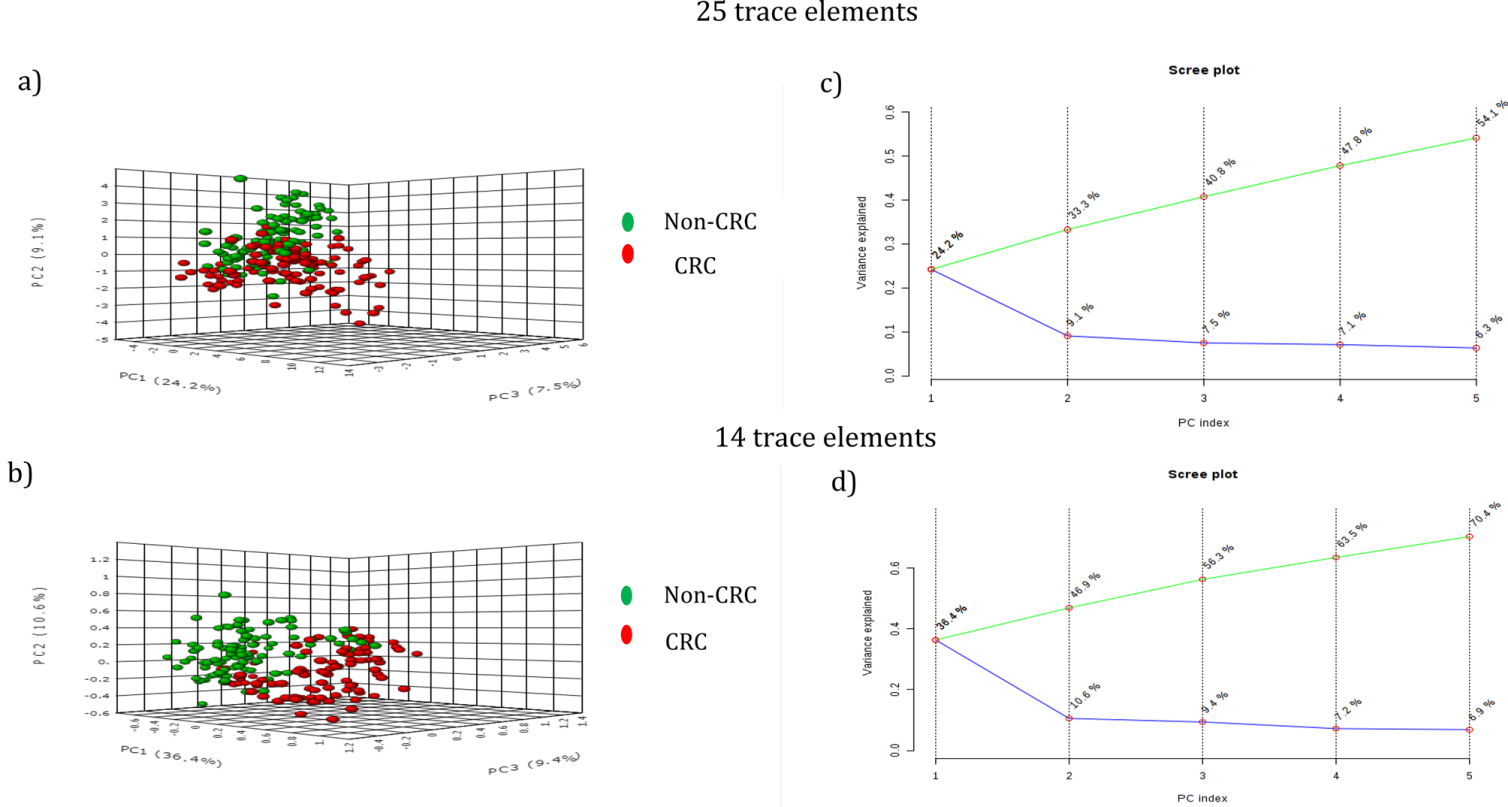
Trace elements distribution. (**a**) PCA showed a distribution of 25 trace elements in the CRC and non-CRC group in the adjacent cluster and can be distinguished by the three main components. (**b**) PCA shows a clear cluster for CRC and non-CRC group based on the distribution of 14 significant trace elements (**c**) Plot scree shows the variance explained with the three main components of the 25 trace elements is 40.8% while the addition to the five components can improve the explained of the variance to 54.1%. (**d**) Plot scree shows the variance explained with the three main components of 14 trace elements is 56.3% while adding to five components can increase the variance explained to 70.4%.

Our results showed a clear alteration of the TE levels in CRC samples compared to non-CRC samples. The correlation between the 25 TEs were observed more in CRC patients (Table 1), with a clear clustering formed between CRC and non-CRC samples. The linkage analysis showed three clusters with a distance of 10–15 in the CRC group, and two clusters in the non-CRC group (Fig. 2). The essential TEs with antioxidant function, i.e., Se and Zn, clustered together in the non-CRC group but were in different clusters in the CRC group. PCA using 14 selected TEs clustered the CRC patients in a clear grouping compared to the non-CRC patients, and this clustering performed better than using the 25 TEs (Fig. 3). The variance for the 14 TEs improved to 56.3% for the three main components compared to the 40.8% from 25 TEs. However, it increased to 70.4% for the five main components compared to 54.1% for 25 TEs.

**Table 1:** Comparison of trace elements correlation (correlation coefficient, r ≥ 0.5) between CRC and non-CRC groups.

Correlation	CRC		Non CRC	
	r value	*p* value	r value	*p* value
Ba–Ga	0.68	< 0.001	0.56	< 0.001
Ag–Ga	0.67	< 0.001	0.57	< 0.001
Ga–Mn	0.61	< 0.001	0.72	< 0.001
Ba–Ag	0.58	< 0.001	0.61	< 0.001
Ni–Cr	0.57	< 0.001	0.65	< 0.001
Ba–Sr	0.57	< 0.001	0.60	< 0.001
Mn–Li	0.55	< 0.001	0.63	< 0.001
U–Ag	0.50	< 0.001	0.65	< 0.001
Ba–Cs	0.68	< 0.001	NS	
Cs–Ga	0.68	< 0.001	NS	
U–Li	0.66	< 0.001	NS	
Cs–Ag	0.64	< 0.001	NS	
Ba–Mn	0.63	< 0.001	NS	
U–Ba	0.62	< 0.001	NS	
Ag–Sr	0.61	< 0.001	NS	
Cs–Sr	0.60	< 0.001	NS	
Pb–TI	0.60	< 0.001	NS	
Pb–Al	0.59	< 0.001	NS	
Ba–Li	0.59	< 0.001	NS	
Sr–Ga	0.58	< 0.001	NS	
Ga–Be	0.56	< 0.001	NS	
Ag–Be	0.56	< 0.001	NS	
Mn–Al	0.54	< 0.001	NS	
Cs–Be	0.53	< 0.001	NS	
Cs–Mn	0.52	< 0.001	NS	
TI–Al	0.52	< 0.001	NS	
Cs–V	0.52	< 0.001	NS	
Pb–C0	0.52	< 0.001	NS	
V–Be	0.51	< 0.001	NS	
U–Ga	0.51	< 0.001	NS	
U–Mn	0.51	< 0.001	NS	
Ba–Be	0.51	< 0.001	NS	
U–Sr	0.50	< 0.001	NS	
Ag–Mn	0.50	< 0.001	NS	
V–Al	0.50	< 0.001	NS	
Ba–Al	NS		0.81	< 0.001
Ni–Al	NS		− 0.67	< 0.001
Ga–Li	NS		0.64	< 0.001
Cr–Al	NS		− 0.60	< 0.001
Ba–Ni	NS		− 0.60	< 0.001
Ag–Al	NS		0.56	< 0.001
Ga–Al	NS		0.56	< 0.001
Cr–Mg	NS		0.55	< 0.001
Sr–Mn	NS		0.54	< 0.001
Sr–Rb	NS		0.52	< 0.001
U–TI	NS		0.51	< 0.001

### Serum TEs as biomarkers

The levels of the 14 significant TEs were analysed further to obtain the respective cut-off screening values for CRC (Table 2). The cut-off points obtained were compared with the values from the Agency for Toxic Substances and Disease Registry (ATSDR). From the comparison, only Be and As were within the normal range by the ATSDR but not the others. Most of the TE levels by the ATSDR were measured using AAS (atomic absorption spectrometry) and the reference values were derived from mainly the Western population. Only Be and Zn had AUC values ≥ 0.80; therefore, the subsequent analysis for determining TEs as biomarkers used the ratio of Be and Zn with the other 12 TEs. The Co/Zn ratio had the highest AUC, followed by the ratios of Be/Zn (0.86) and Rb/Zn (0.85) (Table 2).

**Table 2:** Evaluation of single/ratio trace elements as a biomarker for CRC and comparison with the reported normal range.

Trace elements	Suggested cut off value (ug/L)	AUC ( 95CI)	*p* value	Reported normal range (ug/L)	Reference
Li	3633.62	0.60 (0.52–0.68)	0.016	NA	NA
Be	0.93	0.80 (0.75–0.86)*	< 0.001	0.28–1.00	ATSDR 2002
Al	95.02	0.73 (0.65–0.80)	< 0.001	1.00–3.00	ATSDR 2011
Co	4.77	0.72 (0.65–0.79)	< 0.001	5.70–7.90	ATSDR 2004
Cu	1892.77	0.59 (0.51–0.67)	0.026	2390.00–3460.00	ATSDR 2004
Zn	1103.06	0.83 (0.77–0.89)*	< 0.001	1000	ATSDR 2005
As	0.94	0.64 (0.60–0.72)	< 0.001	< 1.00	ATSDR 2007
Se	81.74	0.66 (0.58–0.73)	< 0.001	125	ATSDR 2003
Rb	12.63	0.67 (0.60–0.74)	< 0.001	NA	NA
Cd	0.19	0.71 (0.64–0.78)	< 0.001	0.31	ATSDR 2012
Ba	151.03	0.66 (0.58–0.73)	< 0.001	NA	NA
Hg	0.76	0.62 (0.54–0.70)	0.003	0.5	ASTDR 1999
TI	0.41	0.63 (0.56–0.71)	0.001	NA	NA
Pb	0.53	0.65 (0.58–0.73)	< 0.001	15	ASTDR 2007

Single/ratio trace elements	AUC (95CI)	Sensitivity	Specificity	PPV	NPV
Co/Zn	0.87 (0.82–0.92)	79.4	76.5	75.9	79.1
Be/Zn	0.86 (0.81–0.91)	77.5	75.5	75.2	76.8
Rb/Zn	0.85 (0.80–0.90)	76.5	73.5	74.8	79.6
Cd/Zn	0.84 (0.78–0.90)	79.4	76.5	70	83.1
Zn	0.83 (0.77–0.89)	80.4	77.5	79.8	78.1
Cu/Zn	0.83 (0.77–0.88)	76.5	73.5	74.3	75.8
Pb/Zn	0.83 (0.77–0.88)	77.5	74.5	75	74
As/Zn	0.81 (0.76–0.86)	77.5	74.5	75	76
Al/Zn	0.80(0.74–0.86)	77.5	74.5	75.2	76.8
Be	0.80(0.75–0.86)	67.6	77.5	75	70.5

*AUC* area under curve, *PPV* positive predictive value, *NPV* negative predictive value.

### The interaction between serum TE levels and environmental factors

CRC patients with red meat intake ≥ 50 g/day showed the highest contribution of risk due to interaction with Co ≥ 4.77 µg/L followed by Zn < 1103.06 µg/L and Al ≥ 95.02 µg/L. After controlling the confounder factors, the interactions contributed 97% (Co), 95% (Zn), and 88% (Al) of CRC risk (Table 3). Only Zn < 1103.06 µg/L with white meat intake < 50 g/day showed positive interaction through multiplicative calculation in determining CRC risk, which was 39 times. The interaction between obesity with Co ≥ 4.77 µg/L or Zn < 1103.06 µg/L contributed to increased CRC risk of 88% and 65%, respectively.

**Table 3:** Interaction analysis between red meat intake with Zn, Co and Al levels.

Trace elements	Red meat intake	CRC	Non-CRC	Univariate^a^					Adjusted^b^				
(ug/L)	(g/day)	n	n	OR	(95% CI)	Interaction	ORINT	ORINT 95%CI	OR	95%CI	Interaction	ORINT	ORINT 95%CI
Zn ≥ 1103.06	−	6	66	1		Multiplicative	5.49	(3.48, 8.65)	1		Multiplicative	0.53	(0.04, 7.34)
Zn < 1103.06	−	22	16	15.13	(5.27, 43.44)	RERI	0.27	(0.03, 0.50)	25.87	(4.49, 149.10)	RERI	0.22	(− 0.13, 0.57)
Zn ≥ 1103.06	+	18	16	12.37	(4.23, 36.19)	AP	0.92	(0.83, 1.01)	16.8	(1.02, 102.89)	AP	0.95	(0.89, 1.03)
Zn < 1103.06	+	56	4	154	(41.37, 573.21)				150.35	(5.51, 4103.67)			
Co < 4.77	−	14	68	1		Multiplicative	5.6	(3.45, 9.09)	1		Multiplicative	0.72	(0.04, 12.16)
Co ≥ 4.77	−	14	14	4.86	(1.90, 12.41)	RERI	0.49	(0.06, 0.92)	8.63	(1.58, 47.09)	RERI	0.52	(− 0.42, 1.46)
Co < 4.77	+	29	16	8.8	(3.81, 20.36)	AP	0.89	(0.79, 0.98)	25.93	(2.21, 303.90)	AP	0.97	(0.91, 1.03)
Co ≥ 4.77	+	45	4	54.64	(16.90, 176.64)				55.5	(3.98, 773.62)			
Al < 95.02	−	6	60	1		Multiplicative	6.6	(4.01, 10.83)	1		Multiplicative	72.76	(0.55, 9673.37)
Al ≥ 95.02	−	22	22	10	(3.58, 27.91)	RERI	0.25	(0.03, 0.47)	10.69	(1.73, 66.13)	RERI	0.21	(− 0.11, 0.53)
Al < 95.02	+	18	19	9.47	(3.29, 27.30)	AP	0.89	(0.78, 1.01)	28.34	(1.50, 536.77)	AP	0.88	(0.68, 1.09)
Al ≥ 95.02	+	56	1	560	(65.35, 4798.38)				32.97	(1.93, 562.08)			

*OR* odds ratio, *ORINT* odds ratio due to interaction.

^a^Not adjusted to other factors, ^b^Adjusted to all environmental risk factors.

### Development of Risk Prediction Model for CRC (CRC RPM)

#### CRC RPM for high and low CRC risk

We used two datasets for developing the CRC RPM using TE levels, environmental risk factors, and the TE–environmental risk factor combination (Table 4). The ANN algorithm analysis of the training data (n = 159) yielded higher values for accuracy, sensitivity, specificity, PPV, and NPV for the CRC RPM using a panel of the 14 TEs. For CRC RPM using environmental risk factors, the SVM algorithm determined higher accuracy for the training data (83.0%), followed by the results using the ANN algorithm (79.8%). However, with the test data, the SVM and ANN algorithms determined 10% and 2% accuracy, respectively, for the CRC RPM. Therefore, the lower RMSE (root mean square error) value was required to select the best algorithm for the CRC RPM. The ANN algorithm determined a low RMSE value for the RPM using environmental risk factors. The best algorithm for the CRC RPM for the TE–environmental risk factor combination was LR. This model had the highest accuracy and AUC value compared to using the 14 TEs or environmental factors alone.

**Table 4:** Development of CRC RPM (high and low risk) using trace element, environmental risk factors and a combination of trace element-environmental risk factors.

Evaluation criteria	Trace element-based						Environmental factor-based						Trace element-based & environmental factor-based					
	Training data (n = 159)			Test data (n = 40)			Training data (n = 159)			Test data (n = 40)			Training data (n = 159)			Test data (n = 40)		
	SVM	ANN	LR	SVM	ANN	LR	SVM	ANN	LR	SVM	ANN	LR	SVM	ANN	LR	SVM	ANN	LR
Correctly classified	146	157	147	39	37	37	132	127	126	29	31	30	155	157	159	39	38	39
Incorrectly classified	13	2	12	1	3	3	27	32	33	11	9	10	4	2	0	1	2	1
Accuracy (%)	91.8	98.7	92.5	97.5	92.5	92.5	83.0	79.8	79.3	72.5	77.5	75.0	97.5	98.7	100.0	97.5	95.0	97.5
Sensitivity	0.89	0.99	0.92	1.00	0.95	1.00	0.85	0.83	0.81	0.85	0.79	0.81	1.00	0.98	1.00	1.00	0.91	0.95
Specificity	0.94	0.99	0.93	0.95	0.90	0.87	0.81	0.77	0.78	0.67	0.76	0.71	0.95	1.00	1.00	0.95	1.00	1.00
PPV	0.95	0.99	0.94	0.95	0.90	0.85	0.82	0.77	0.78	0.55	0.75	0.65	0.95	1.00	1.00	0.95	1.00	1.00
NPV	0.88	0.99	0.91	1.00	0.95	1.00	0.84	0.83	0.81	0.90	0.80	0.85	1.00	0.97	1.00	1.00	0.90	0.95
Variable	14	14	14	14	14	14	6	6	6	6	6	6	20	20	20	20	20	20
Kappa Statistic	0.84	0.97	0.85	0.95	0.85	0.85	0.66	0.60	0.59	0.45	0.55	0.50	0.95	0.97	1.00	0.95	0.90	0.95
RMSE	NA	NA	NA	0.0045	0.0034	0.0046	NA	NA	NA	0.0041	0.0039	0.0039	NA	NA	NA	0.005	0.0047	0.0035
AUC	0.98	0.99	0.98	1.00	0.98	0.99	0.90	0.84	0.87	0.78	0.87	0.88	0.99	1.00	1.00	1.00	1.00	0.99

*SVM* support vector machine, *ANN* artificial neural network, *LR* logistic regression, *RMSE* root mean square error, *PPV* positive predictive value, *NPV* negative predictive value.

**Figure 4:**
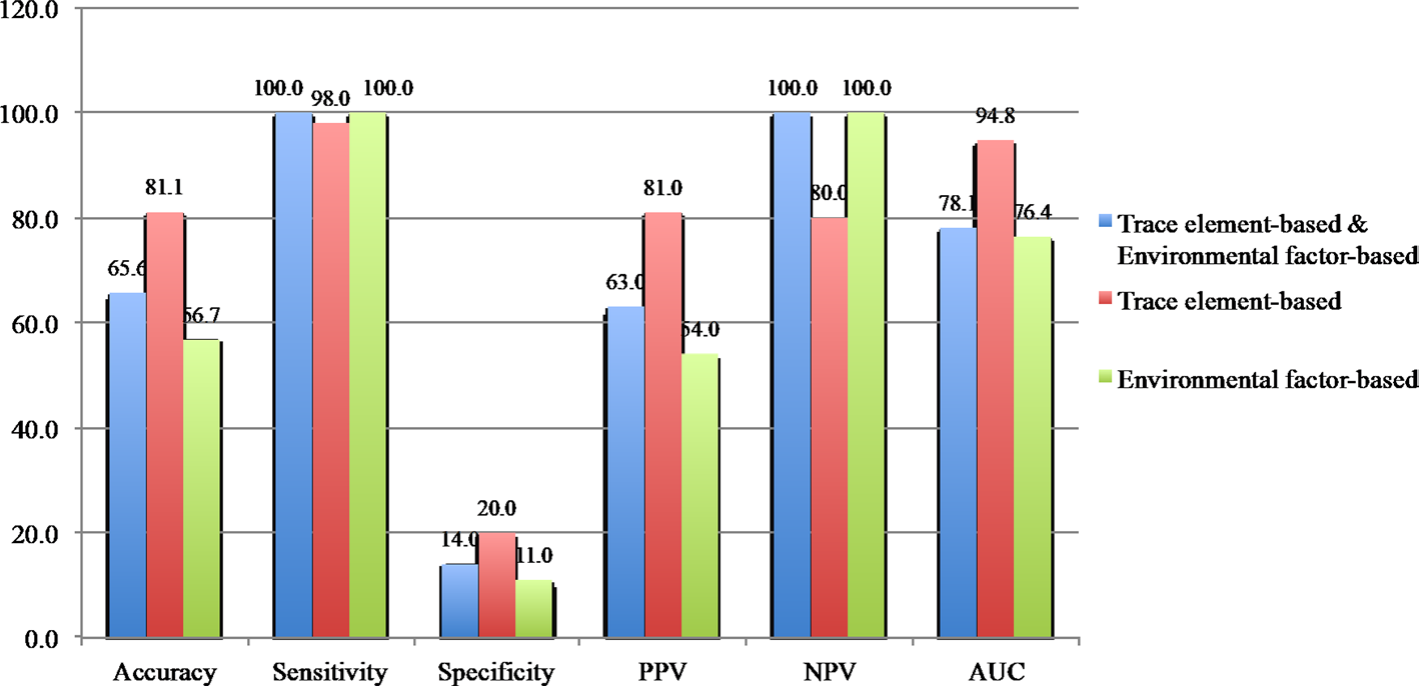
Validation of CRC RPM among ASX CRC (n = 90).

The CRC RPM was further tested with the ASX CRC cases and showed that the 14-TE panel (81.1%) was the best model (Fig. 4). Although the RPM using the 14-TE panel yielded higher accuracy and AUC value, it was not specific. The RPM analysis showed that the 14-TE panel could predict CRC risk among the asymptomatic population.

#### CRC RPM for high, moderate, and low CRC risk

The CRC RPM developed in the discovery phase showed good accuracy in predicting high and low CRC risk. However, the accuracy decreased when tested with ASX CRC samples. This might have been because the data used in RPM development were from patients with CRC, hence reflecting a late stage pathology as compared to the asymptomatic stage. Therefore, the CRC RPM required improvement with the inclusion of data from ASX CRC itself. The improved CRC RPM could evaluate CRC risk simultaneously into three levels: high (CRC), moderate (ASX CRC), and low (non-CRC). Before the new CRC RPM was developed, selection of variables for the TEs and environmental risk factors was required that could differentiate the CRC, ASX CRC, and non-CRC groups. Only the selected variables were included in the CRC RPM development. The level of 24 TEs (Ag, Al, As, Ba, Be, Cd, Co, Cr, Cs, Cu, Ga, Li, Mg, Mn, Ni, Pb, Rb, Se, Sr, Tl, U, V, Zn, Hg) and eight environmental risk factors (age, ethnicity, comorbidities, smoking status, physical activity, obesity, red meat intake, white meat intake) were significantly different between the three groups and were included in model development.

The CRC RPM using the 24-TE panel produced the highest accuracy (100%) after testing with the test data (Table 5). This was followed by the CRC RPM using the TE–environmental risk combination (86.5%) and environmental risk factors alone (67.3%). Besides that, the LR algorithm was selected for all three CRC RPMs, with the training data yielding high accuracy. Although the SVM algorithm yielded higher accuracy for the CRC RPM using environmental risk factors as compared to LR, the accuracy decreased by almost 25% for the SVM algorithm as compared to LR (7.1%). Therefore, LR was selected for the CRC RPM using environmental risk factors.

**Table 5:** Development of CRC RPM (high, moderate and low risk) using trace element, environmental risk factors and a combination of trace element-environmental risk factors.

Evaluation Criteria	Trace element-based						Environmental factor-based						Trace element-based & Environmental factor-based					
	Training data (n = 168)			Test data (n = 52)			Training data (n = 168)			Test data (n = 52)			Training data (n = 168)			Test data (n = 52)		
	SVM	ANN	LR	SVM	ANN	LR	SVM	ANN	LR	SVM	ANN	LR	SVM	ANN	LR	SVM	ANN	LR
Correctly classified	161	141	168	46	39	47	142	110	125	31	32	35	168	157	168	42	45	45
Incorrectly classified	7	27	0	6	13	5	26	58	43	21	20	17	0	11	0	10	7	7
Accuracy (%)	95.8	84.0	100.0	88.5	75.0	100.0	84.5	65.5	74.4	59.6	61.5	67.3	100.0	93.5	100.0	80.8	86.5	86.5
**High risk (CRC)**																		
Sensitivity	0.97	0.82	1.00	0.85	0.70	1.00	0.84	0.70	0.69	0.50	0.65	0.65	1.00	1.00	1.00	0.75	1.00	0.85
Specificity	0.97	0.89	1.00	0.94	0.81	1.00	0.93	0.71	0.86	0.78	0.72	0.78	1.00	0.94	1.00	0.88	0.81	0.91
PPV	0.95	0.81	1.00	0.89	0.70	1.00	0.86	0.58	0.74	0.59	0.59	0.65	1.00	0.90	1.00	0.79	0.77	0.85
NPV	0.98	0.90	1.00	0.91	0.81	1.00	0.91	0.81	0.83	0.71	0.77	0.78	1.00	1.00	1.00	0.85	1.00	0.91
**Moderate risk (ASX CRC)**																		
Sensitivity	0.98	0.92	1.00	1.00	0.04	1.00	0.88	0.59	0.82	0.64	0.45	0.73	1.00	0.86	1.00	1.00	0.82	0.91
Specificity	0.97	0.97	1.00	0.98	0.72	1.00	0.93	0.94	0.91	0.88	0.90	0.90	1.00	1.00	1.00	0.93	1.00	0.98
PPV	0.93	0.92	1.00	0.92	0.04	1.00	0.85	0.81	0.81	0.58	0.56	0.67	1.00	1.00	1.00	0.79	1.00	0.91
NPV	0.99	0.97	1.00	1.00	0.67	1.00	0.95	0.84	0.92	0.90	0.86	0.93	1.00	0.94	1.00	1.00	0.95	0.98
Variable	24	24	24	24	24	24	8	8	8	8	8	8	32	32	32	32	32	32
Kappa Statistic	0.94	0.76	1.00	0.82	0.61	0.85	0.77	0.48	0.62	0.38	0.40	0.50	1.00	0.90	1.00	0.71	0.79	0.79
AUC	0.98	0.99	1.00	0.99	0.86	1.00	0.88	0.88	0.90	0.86	0.77	0.83	1.00	0.97	1.00	0.88	0.88	0.94

*SVM* support vector machine, *ANN* artificial neural network, *LR* logistic regression, *RMSE* root mean square error, *PPV* positive predictive value, *NPV* negative predictive value.

**Figure 5:**
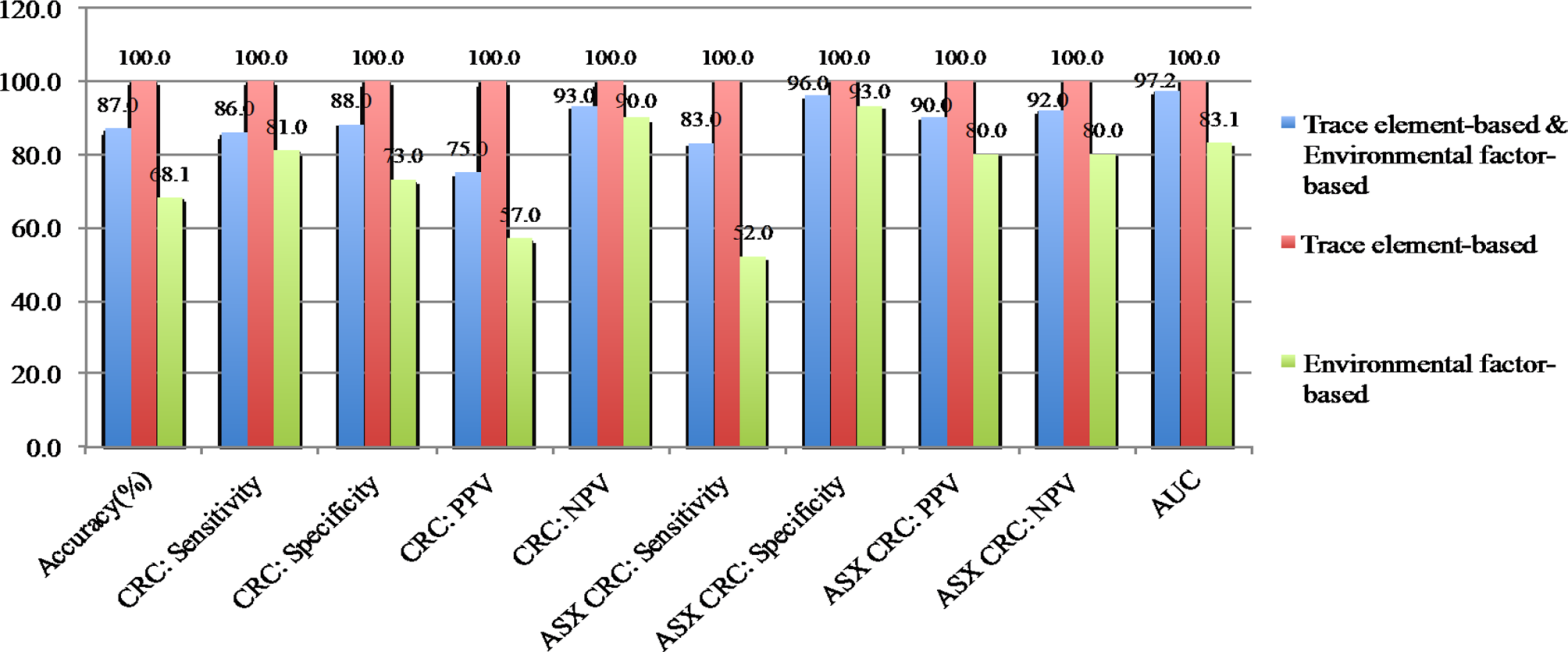
Validation of CRC RPM using validation data (n = 69).

CRC RPM accuracy was evaluated using the validation data (n = 69). The highest accuracy for the CRC RPM was based on the 24-TE panel (Fig. 5). The findings confirm the model’s consistency in predicting CRC risk with good accuracy, sensitivity, specificity, PPV, NPV, and AUC value. It showed that the CRC RPM could perform accurate predictions using the 24-TE panel compared to RPMs using the TE–environmental risk factor combination or environmental risk factors alone. The CRC RPM was not only able to predict high CRC risk in individuals, but also among individuals with moderate CRC risk and who did not have any CRC symptoms.

## Discussion

### Determination, comparison, and classification of TE levels between CRC and non-CRC cases

In the present study, we identified a panel of 14 TEs (Li, Be, Al, Co, Cu, Zn, As, Se, Cd, Rb, Ba, Hg, Tl, Pb) that separated CRC from the non-CRC samples. We noted that 10 of the 14 TEs (Li, Be, Al, Co, Rb, Ba, As, Hg, Tl, Pb) have not been reported to be altered in patients with CRC. Be, Al, Co, Rb, Ba, As, Hg and Pb have been reported for other cancer types, but not for CRC^17–24^. High or low levels of TEs can possibly contribute to CRC through various mechanisms. Among the mechanisms that have been reported is inhibition of DNA repair, inhibition of DNA methylation, increased oxidative stress, and altered gene expression^25^.

We found that the levels of Se, Zn, and Cd are in concordance with the results of a previous study on patients with CRC^26–28^. The low levels of Se and Zn we found in the patients with CRC have also been reported previously. It is believed that Se acts through an antioxidant defence system to reduce oxidative stress and minimise DNA damage^29^. Similar to Se, Zn is an important co-factor in antioxidant enzymes (superoxide dismutase [SOD], GPx) and is involved in the defence systems of the body^30^. In vivo and in vitro studies have proven that Zn can prevent cancer development through apoptosis mechanisms^31^. High Cd levels in serum^32^ and tissue^33^ have also been reported in patients with CRC. Cd is also a heavy metal and has been categorised as a human carcinogen^34^. The mechanisms involved in CRC formation are through oncogene activation and the inhibition of apoptosis^35^.

For Cu, our findings were inconsistent when compared to previous studies. We found that patients with CRC had high Cu levels, but Milde et al.^36^ found that such patients had low Cu levels. The difference may be due to sample size, where Milde et al. studied only 20 patients, while our study involved 102 patients. Other researchers have reported similar findings to ours, where CRC patients with Dukes’ stage C and D had higher Cu levels^37^. In the present study, most patients with CRC were diagnosed at Dukes’ stage C. This indirectly explains why the patients with CRC in the present study had high Cu levels compared to the patients without CRC. Khoshdel et al.^38^ reported the same finding in a large sample of patients with CRC from Iran (n = 119), but unlike the present study using ICPMS, they used AAS. Therefore, the difference in Cu level findings in patients with CRC should be investigated further in a different and larger cohort of samples.

We found that there was more correlation between TEs and CRC than with non-CRC cases. Ba, Cs, Ga, U, Li, Ag, Mn, U, Sr, Pb, Tl, Sr, Be, Al, V, and Co had positive correlation values > 0.5. These TEs were all present in high quantities in the patients with CRC compared to the values recommended by the accredited ATSDR. When there is a correlation between TEs, especially at high levels, it is likely to have a toxic effect on the human body and thus could lead to CRC formation^39^.

Patients with CRC also have disrupted TE distribution, especially for essential TEs such as Cu, Zn, and Se. These TEs were grouped into different clusters in patients with CRC patients compared to non-CRC cases. Feng et al.^40^ studied patients with breast cancer and found that these three TEs are closely related to the status of oxidative stresses that can contribute to cancer formation. There are few findings on the correlation between TEs and their distribution patterns in CRC. However, there is a significant relationship between the TEs and their distribution in patients with CRC as compared to patients without CRC.

Biomarkers using TEs attracted more attention following the reporting of evidence from previous studies on TEs and disease risk^41^. TEs have been used for differentiating to patients with and without cancer such as in breast cancer^42^, lung cancer^43^, prostate cancer^44^, and CRC^45^. Although TEs have attracted a lot of attention as potential cancer biomarkers, the cut-off values of the respective TEs have not been determined. Reference sources remain scarce, and the normal level values ​​are typically referred through the ATSDR website (https://www.atsdr.cdc.gov/). The TE values ​​on the ATSDR website are more relevant to the general population rather than to respective diseases including cancers. Moreover, the reference values were established in the last decade based on the Western population. Therefore, a screening cut-off value for patients with CRC itself is much needed to be used for identifying those with high CRC risk. Our findings cut off value for differenting CRC population almost similar with ATSDR value for Be and Zn.

In the present study, though the level of 14 TEs could differentiate CRC and non-CRC samples, only Be and Zn levels had AUC value of ≥ 0.8. The cut-off values ​​for Be and Zn which we have proposed are in the range set by the ATSDR in the general population^46,47^. For the other 12 TEs the AUC values were < 0.8 hence they are less useful as individual biomarkers^48^. Therefore, the cut-off values ​​for Be and Zn we have proposed can be used as reference ​​or screening values for patients with CRC. The cut-off values from this findings may varies with different population but our finding cut off value are in line with ATSDR suggestion in differentiating CRC and non-CRC.

Apart from the individual TE, it has also been suggested that TE ratios can be used as biomarkers. We found that the AUC value can be improved through the use of the TE ratio rather than a single TE. In the present study, the Co/Zn ratio yielded the highest AUC value. However, no study to date has assessed Co or even the Co/Zn ratio as a biological marker for CRC. However, it has been suggested that the Cu/Zn ratio be used as a biomarker, but no AUC or sensitivity values have been specified for the ratio^37^. Although previous studies have focused on the Cu/Zn ratio, our findings on the Co/Zn ratio require further validation of its potential use as a CRC screening test.

### The interaction between serum TEs and environmental factors

We also showed that the interaction between excessive red meat intake with low Zn levels could increase CRC risk. Red meat is a rich source of Zn^49^. Excessive red meat intake increases Zn levels, but its bioavailability depends on homeostasis. Homeostasis is maintained in the gastrointestinal system through the absorption of exogenous Zn, and the secretion and excretion of Zn endogenously^50^. Imbalanced diet, such as food with high-phytate composition (e.g., grains and legumes)^51^ and the presence of certain intestinal microbes^52^ are two examples of factors that can interfere with the effectiveness of Zn homeostasis. This decreases the amount of Zn in the body even with excessive red meat intake. Low Zn levels reduce antioxidant responses for neutralising oxidative stress^53^. Also, the carcinogenic mechanisms of red meat content^54^ can double CRC risk.

Cooking with utensils made from Al or wrapping food in Al foil can cause Al leaching into food^55^. Turhan^56^ showed that Al content was increased by 89–378% if red meat was cooked and wrapped with Al foil. Marinating meat with a mixture of citric acid and lactic acid and wrapping it with Al foil can further enhance the Al content of the meat through leaching^57^. Red meat also has high quantities of Co compared to white meat^58^. The increased Co in red meat can occur through the provision of foods containing high quantities of Co, such as alfalfa seeds or linseed (animal food)^59^. Consequently, excessive red meat consumption indirectly increases Al and Co levels in the human body. The combination of red meat intake with high Al or Co levels stimulates carcinogenic mechanisms in CRC formation^60,61^.

We also showed that the interaction between low intake of white meat (< 50 g/day) and low Zn levels contributed to higher CRC risk. Unbalanced diets^51^ and the presence of certain intestinal microbes^52^ can cause decreased Zn levels due to disturbance of Zn homeostasis. White meat does not produce carcinogens as compared to red meat, but as a result of low Zn levels, oxidative stress remains uncontrolled^62^, increasing CRC risk.

The factor of obesity combined with low Zn levels or high Co levels also increases CRC risk. Zn levels decrease with increased body mass index^63^. Adipose tissue causes systemic changes in the human body, including altering the levels of insulin, insulin-like growth factor-1, leptin, adiponectin, steroids, and cytokines^64^. This can interfere with Zn homeostasis and cause Zn deficiency^65^. In addition, lower levels of a Zn transporter gene, *ZIP14* (SLC39a15), have been reported in obese individuals^66^ and result in Zn reduction in the body^67^. Obesity-induced endocrine changes and gene expression cause low Zn levels. Thus, it can increase oxidative stress and DNA damage^30,68,69^, which further contribute to CRC formation. However, the association between obesity and Co levels remains unknown^70^, as does its relation to the mechanism of disease.

### CRC RPM

We developed RPMs for CRC based on TEs and environmental risk factors. The model was tested on three groups of patients: high-risk (CRC), moderate-risk (ASX CRC), and low-risk (non-CRC). Early in the CRC RPM development, the addition of environmental risk factors to the TEs increased the accuracy of the CRC RPM. However, the accuracy decreased when tested on the ASX CRC group. This may have been due to an inaccuracy of the environmental risk factors information, which relied heavily on the patient’s memory. Hence, the environmental risk factor information obtained is more likely to be biased^71^ than the quantitative measurement of TEs in the patient’s blood.

The 14-TE panel (Li, Be, Al, Co, Cu, Zn, As, Se, Cd, Rb, Ba, Hg, Tl, Pb) could predict high and low CRC risk but was less precise for the moderate risk group. The development of a new CRC RPM using a 24-TE panel (Ag, Al, As, Ba, Be, Cd, Co, Cr, Cs, Cu, Ga, Li, Mg, Mn, Ni, Pb, Rb, Se, Sr, Tl, U, V, Zn, Hg) increased the value of each performance parameter, especially accuracy. This enabled CRC risk assessment to be classified into three categories, i.e., high, medium, and low. This risk stratification method is useful for early detection of patients with high CRC risk^72^. Hence, colonoscopy and tissue biopsy for determining CRC diagnosis may be prioritized to high-risk individuals first, followed by moderate-risk individuals. Early detection of CRC can be performed through this predictive model even if the patient does not show any clinical symptoms of CRC.

To date, there is no CRC RPM using TEs. However, previous studies have shown that TEs can be used to predict the risk of other cancers and diseases. For example, Guo et al.^73^ used a panel of 10 TEs from hair specimens (Mg, P, K, Ca, Cr, Mn, Fe, Cu, Zn, Se) to predict prostate cancer with 95.8% accuracy. In addition, demographic, clinical, and trace elements have been incorporated for predicting Parkinson’s disease^74,75^. This demonstrates the importance of knowledge of the TEs in the human body for use as a predictor of CRC risk. The futher validation may needs from other source population before CRC RPM can be performed in the community.

The main study strength are the novelity of the findings related to TE and less invasive biomarker contribution for early detection of CRC. However, futher validation needs to be done for a more accurate and sensitive results. Information bias is an avoidable situation as we mainly depends on self-reported information. We try to reduce the bias by confirming the self-reported information with family members.

In conclusion, public awareness of healthy and balanced nutrition needs to be improved. Increased awareness of environmental risk factors in the community can reduce the risk of CRC. In Malaysia, various awareness programs have been organised therefore including CRC screening. We would like to recommend the 24-TE panel developed in this study as a screening test for individual stratification with different levels of CRC risk, i.e., high, moderate, or low risk. High-risk individuals should take priority in colonoscopy and tissue biopsy procedures for determining CRC diagnosis, followed by moderate- and low-risk individuals.

## Materials and methods

### Participants

*Discovery Phase.* All participants were newly diagnosed CRC patients from the Universiti Kebangsaan Malaysia (UKM) Medical Centre, Malaysia. Patients were excluded if they had more than one cancer, history or finding of polyps, inflammatory bowel disease (IBD) during colonoscopy, and history of toxic exposure during work. We enrolled 102 patients with CRC and 102 patients without CRC. The participants were interviewed to obtain information on environmental risk factors and underwent blood-taking for TE analysis after histopathology result confirm CRC or not.

#### Validation phase

All participants from The Malaysian Cohort (TMC)^76^ who are diagnosed with CRC during follow up were included as asymptomatic (ASX) CRC. Initial recruitment started in April 2006 through to the end of September 2012. The information on CRC diagnosis was based on self-reporting during follow-up or from mortality data from the Malaysian National Registration Department. Based on information obtained until June 2017, 85 ASX CRC cases were included in this study.

All participants accepted the terms of the study and provided written informed consent. The study was approved by the UKM Medical Research Ethical Committee (FF-2015–380) as following by the guidelines set out in the Declaration of Helsinki.

### Environmental risk factors

All participants completed a set of questionnaires adapted from TMC study, which consisted of information on demographics, socioeconomic status, family history of cancer, comorbidity, smoking status, alcohol consumption, diet intake, body mass index, and physical activity. Diet intake and physical activity were assessed using the food frequency questionnaire and International Physical Activity Questionnaire-Malaysia (IPAQ-M), respectively^77^. The information gather are self-reported and the interview were done by several enumerator. A training session on questionnaire was conducted to minimise the potential interview bias. For the validation phase, the information was extracted from the TMC database.

### Quantification of trace elements

Fasting blood was processed to obtain the serum and stored at − 80 °C until analysis. Samples were pre-treated with acid digestion. The multi-element analysis of 25 TEs (lithium [Li], beryllium [Be], magnesium [Mg], aluminum [Al], vanadium [V], chromium [Cr], manganese [Mn], iron [Fe], cobalt [Co], Ni, copper [Cu], Zn, gallium [Ga], arsenic [As], selenium [Se], rubidium [Rb], strontium [Sr], silver [Ag], cadmium [Cd], cesium [Cs], barium [Ba], mercury [Hg], thallium [Tl], lead [Pb], uranium [U]) was performed using an Agilent 7700 inductively coupled plasma mass spectrometer (ICP-MS)^78^.

### Statistical analysis

Statistical analyses were performed using STATA/SE 13.0, SPSS Modeler version 18, and MetaboAnalyst 4.0. The normality distributions of quantitative data such as TE levels were checked by histogram and the Kolmogorov–Smirnov test. The 25 TEs between the CRC, non-CRC, and ASX CRC samples were compared using the independent *t*-test or analysis of variance for data with a normal distribution; the Mann–Whitney U or Kruskal–Wallis tests were used for data with non-normal distribution. The inter-relationship between each pair of TEs was investigated using Pearson correlation analysis. The distribution pattern of circulating TEs was plotted based on principal component analysis (PCA) and cluster analysis (CA). The best cut-off value for CRC was determined using receiver operating curve (ROC) analysis and the Youden index. The significance level was established at *p* < 0.05.

### Risk Prediction Model for CRC

The Risk Prediction Model (RPM) was developed based on machine learning (ML) algorithms. First, in the CRC RPM for discovery phase, the data were divided into two sets by the partition node of SPSS Modeler for developing a prediction model using three common ML algorithms: logistic regression (LR), support vector machine (SVM), and artificial neural network (ANN). Of the overall data, 80% (n = 159) were used for model development; the remaining 20% (n = 40) were used for model testing. The CRC RPM was validated among the ASX CRC cases (n = 85). Next, an improved CRC RPM with the inclusion of ASX CRC was developed using the same three ML algorithms. The data were divided into three sets: model development, 60% (n = 168); model testing, 20% (n = 52); and model validation, 20% (n = 69).

The independent variables data consisted of different units and therefore required data normalisation. The normalisation was scaled within the range of 0–1^79^. This scaling is suitable for improving the accuracy of numeric computation by the ML algorithms. Accuracy (the percentage of testing data correctly predicted by the model), sensitivity (the proportion of patients with CRC), specificity (the proportion of patients without CRC correctly identified by the model), positive predictive value (PPV), negative predictive value (NPV), and area under the curve (AUC) were used for measuring the performance of the prediction models. Ten-fold cross-validation was used to measure the unbiased estimate of the three prediction models for comparing their performance.

## Supplementary information

Supplementary Information

## Data Availability

All data generated or analysed during this study are included in this published article (and its Supplementary Information files).
