# Author Correction: Delineating colorectal cancer distribution, interaction, and risk prediction by environmental risk factors and serum trace elements

**DOI:** 10.1038/s41598-021-83219-8

**Published:** 2021-02-23

**Authors:** Azmawati Mohammed Nawi, Siok Fong Chin, Luqman Mazlan, Rahman Jamal

**Affiliations:** 1 Department of Community Health, Faculty of Medicine, UKM Medical Center, Universiti Kebangsaan Malaysia, Jalan Yaacob Latif, Bandar Tun Razak, 56000 Cheras, W. Persekutuan Malaysia; 2 Medical Molecular Biology Institute (UMBI), Universiti Kebangsaan Malaysia (UKM), Jalan Yaacob Latif, Bandar Tun Razak, 56000 Cheras, W. Persekutuan Malaysia; 3 Department of Surgery, UKM Medical Center, UKM, Cheras, Malaysia

Correction to: *Scientific Reports* http://doi.org/10.1038/s41598-020-75760-9, published online 29 October 2020

In the original version of this Article, authors Siok Fong Chin and Rahman Jamal were incorrectly affiliated with “Department of Community Health, Faculty of Medicine, UKM Medical Center, Universiti Kebangsaan Malaysia, Jalan Yaacob Latif, Bandar Tun Razak, 56000, Cheras, W. Persekutuan, Malaysia.”

The correct affiliation for Siok Fong Chin and Rahman Jamal is listed below.

Medical Molecular Biology Institute (UMBI), Universiti Kebangsaan Malaysia (UKM), Jalan Yaacob Latif, Bandar Tun Razak, 56000, Cheras, W. Persekutuan, Malaysia

This error has now been corrected in the PDF and HTML versions of the Article.

