# Supplementary material for: Delineating colorectal cancer distribution, interaction, and risk prediction by environmental risk factors and serum trace elements
Source: Sci Rep. 2020 Oct 29;10:18670. doi: 10.1038/s41598-020-75760-9 (PMC7596468; doi:10.1038/s41598-020-75760-9)
Supplement: Supplementary file 1 — Supplementary Information [file 41598_2020_75760_MOESM1_ESM.docx]

Supp. 1 Environmental risk factors among CRC and non-CRC patients

| **Environmental Factors** | **Discovery Phase** | |  |  |
| --- | --- | --- | --- | --- |
|  | **CRC** | **Non CRC** | **X^2^/t value** | **p value** |
|  | **n (%)** | **n (%)** |  |  |
|  | **n=102** | **n=102** |  |  |
| **Non-modifiable** |  |  |  |  |
| Age (years)# | 63.16(10.33) | 60.37 (10.70) | 1.89^a^ | 0.060 |
| Gender |  |  | 4.44^b^ | 0.035* |
| Male | 63 (61.8) | 48 (47.1) |  |  |
| Female | 39 (38.2) | 54 (52.9) |  |  |
| Ethnic |  |  | 2.92^b^ | 0.232 |
| Malay | 62 (60.8) | 50 (49.0) |  |  |
| Chinese | 33 (32.4) | 44 (43.1) |  |  |
| Indian | 7 (6.9) | 8 (7.8) |  |  |
| Working |  |  | 0.97 | 0.325 |
| Yes | 52(51.0) | 59(57.8) |  |  |
| No | 50(49.0) | 43(42.2) |  |  |
| Comorbidity |  |  | 0.74^b^ | 0.390 |
| Yes | 59 (57.8) | 65 (63.7) |  |  |
| No | 43 (42.2) | 37 (36.3) |  |  |
| Family history of CRC |  |  | 0.13^b^ | 0.713 |
| Yes | 19 (18.6) | 17 (16.7) |  |  |
| No | 83 (81.4) | 85 (83.3) |  |  |
| **Modifiable** |  |  |  |  |
| Smoking |  |  | 8.02^b^ | <0.001* |
| Yes | 49(48.0) | 15(14.7) |  |  |
| No | 53( 52.0) | 87( 85.3) |  |  |
| Alcohol Consumption |  |  | 0.00^b^ | 1.000 |
| Yes | 5 (4.9) | 5 (4.9) |  |  |
| No | 97 (95.1) | 97 (95.1) |  |  |
| Body mass index |  |  | 15.40^b^ | <0.001* |
| Normal | 34 (33.4) | 61 (59.9) |  |  |
| Overweight | 45 (44.1) | 23 (22.5) |  |  |
| Obese | 23 (22.5) | 18 (17.6) |  |  |
| Physical Activity |  |  | 27.81^b^ | <0.001* |
| Low | 61 (59.8) | 24 (23.5) |  |  |
| Moderate | 15 (14.7) | 32 (31.4) |  |  |
| High | 26 (25.5) | 46 (45.1) |  |  |
| History of diet intake |  |  |  |  |
| Red meat |  |  | 35.44^b^ | <0.001* |
| ≥ 50g/day | 58 (56.9) | 17 (16.7) |  |  |
| < 50g/day | 44 (43.1) | 85 (83.3) |  |  |
| White meat |  |  | 6.41^b^ | 0.011* |
| ≥ 50g/day | 47 (46.1) | 65 (63.7) |  |  |
| < 50g/day | 55 (53.9) | 37 (36.3) |  |  |
| Fibre |  |  | 0.76^b^ | 0.382 |
| ≥ 10g/day | 68 (66.7) | 62 (60.8) |  |  |
| < 10g/day | 34 (33.3) | 40 (39.2) |  |  |
| Dietary intake of trace elements^ |  |  |  |  |
| Zn(mg) | 6.12 (2.82-7.69) | 6.74 (3.75-9.18) | 3868.50^c^ | 0.051 |
| Cu(mg) | 1.52 (1.20-1.89) | 1.55 (1.38-2.13) | 2519.30^c^ | 0.271 |
| Se( µg) | 83.13 (70.14-89.36) | 87.56 (83.17-91.45) | 4102.10^c^ | 0.247 |
| Mg( mg) | 57.04 (39.82-75.66) | 54.47 (37.41-79.73) | 5089.50^c^ | 0.79 |
| Fe(mg) | 12.92 (8.33-17.92) | 11.61 (7.08-14.40) | 4613.50^c^ | 0.163 |
| ^a^  Independent t-test ^b^ Chi square test, ^c^ Mann Whitney test * Significance difference at p value < 0.05 | | | | |
| # Means(SD), ^Median (Interquartile range, IQR) | |  |  |  |

Supp. 2 Comparison of 14 significant trace element levels with previous studies related to CRC and others cancer

| **Trace element** | **Value(µg/L ) In this study CRC=102** | **CRC (Reference)** | **Others cancer (Reference)** |
| --- | --- | --- | --- |
| Li | ↑ | N | N |
| Tl | ↑ | N | N |
| Be | ↑ | N | ↑ Lung cancer (Hollins et al., 2009) |
| Al | ↑ | N | ↑ Breast cancer (Romanowicz-Makowska et al., 2011) |
| Co | ↑ | N | ↑ Lung cancer (Moulin et al., 1998) |
| Rb | ↑ | N | ↑ Breast cancer (Su et al., 2011) |
| As | ↑ | N | ↑ Bladder cancer (Tsuji et al., 2014) |
| Ba | ↑ | N | ↑ Breast cancer (Blaurock-Busch et al., 2014) |
| Hg | ↑ | N | ↑ Renal cancer (Sá et al., 2016) |
| Pb | ↑ | N | ↑ Breast cancer (Mardanshahi & Shokrzadeh , 2017; McElroy et al., 2008) |
| Zn | ↓ | ↓ Gupta et al. (1993); Milde et al. (2001) | ↓ Breast cancer (Kaczmarek et al. 2012; Wu et al. 2015) |
| Se | ↓ | ↓ Milde et al. (2001) | ↓ Larynx cancer (Lubiński et al. 2018), Breast cancer (Hashemi et al. 2017) |
| Cd | ↑ | ↑ Gupta et al. (1993), Klimczak et al. (2016) | ↑ Endometrial cancer (McElroy et al. 2017), Prostate cancer (Abhishek et al. 2017) |
| Cu | ↑ | ↓ Milde et al. (2001),↑ Ribeiro et al. (2016b) | ↑ Solid and blood cancer (Gupte & Mumper 2009) |
| N No report, ↑ Higher from control, ↓ Lower from control | | | |

N No report, ↑ Higher from control, ↓ Lower from control
